# Ectopic Expression of AID in a Non-B Cell Line Triggers A∶T and G:C Point Mutations in Non-Replicating Episomal Vectors

**DOI:** 10.1371/journal.pone.0001480

**Published:** 2008-01-23

**Authors:** Tihana Jovanic, Benjamin Roche, Géraldine Attal-Bonnefoy, Olivier Leclercq, François Rougeon

**Affiliations:** Unité de Génétique et Biochimie du Développement, Département d'Immunologie, URA CNRS 2581, Institut Pasteur, Paris, France; National Institute on Aging, United States of America

## Abstract

Somatic hypermutation (SHM) of immunoglobulin genes is currently viewed as a two step process initiated by the deamination of deoxycytidine (C) to deoxyuridine (U), catalysed by the activation induced deaminase (AID). Phase 1 mutations arise from DNA replication across the uracil residue or the abasic site, generated by the uracil-DNA glycosylase, yielding transitions or transversions at G:C pairs. Phase 2 mutations result from the recognition of the U∶G mismatch by the Msh2/Msh6 complex (MutS Homologue), followed by the excision of the mismatched nucleotide and the repair, by the low fidelity DNA polymerase η, of the gap generated by the exonuclease I. These mutations are mainly focused at A∶T pairs. Whereas in activated B cells both G:C and A∶T pairs are equally targeted, ectopic expression of AID was shown to trigger only G:C mutations on a stably integrated reporter gene. Here we show that when using non-replicative episomal vectors containing a GFP gene, inactivated by the introduction of stop codons at various positions, a high level of EGFP positive cells was obtained after transient expression in Jurkat cells constitutively expressing AID. We show that mutations at G:C and A∶T pairs are produced. EGFP positive cells are obtained in the absence of vector replication demonstrating that the mutations are dependent only on the mismatch repair (MMR) pathway. This implies that the generation of phase 1 mutations is not a prerequisite for the expression of phase 2 mutations.

## Introduction

Affinity maturation of the humoral immune response arises from the stepwise introduction of single nucleotide substitutions into the variable regions of immunoglobulin genes during B cell proliferation in germinal centers. This process is known as somatic hypermutation (SHM) and depends on the expression of AID, the activation induced cytidine deaminase whose expression is restricted to centroblast B cells [1], [2].

Analysis of the altered mutation pattern in mice deficient in MSH2, a mismatch repair (MMR) protein, led to the proposal that SHM is a two step process. SHM is initiated by deamination of deoxycytidine (C) to deoxyuridine (U) in single-stranded DNA, produced during the transcription of the variable (V) gene. Phase 1 mutations are introduced during replication across the G:U mismatch and result in G:C to A∶T transitions. If the U base is removed before replication by uracil-DNA glycosylase, the replication of the abasic site, created by a translesion DNA polymerase, gives rise to both transitions and transversions. Phase 2 mutations are mainly restricted to A∶T pairs surrounding a U∶G mismatch and involve the mismatch repair machinery. The recognition of the U∶G mismatch by the Msh2/Msh6 complex results in a mutagenic patch repair mechanism involving exonuclease I and the low-fidelity DNA polymerase η (POLΗ) [3]–[5]. In activated B cells, G:C and A∶T pairs are equally targeted at V genes. However, in B cell lines, as well in non-B cell lines in which AID is ectopically expressed, mutations at G:C pairs are mainly found [6]–[8]; why mutations at A∶T pairs are almost always absent remains unclear. In activated B cells, A∶T mutations are strictly dependent on the Msh2/Msh6 pathway and are presumed to be introduced during patch repair by POLH, in the absence of DNA replication [9], [10]. The function of MMR is to ensure the fidelity of DNA replication by removing mismatches produced during DNA synthesis [11], [12]. The absence of mutations at A∶T pairs in B or non-B cell lines expressing AID could be explained either by the prevalence of phase 1 mutations at G:C pairs preventing MMR from occurring or, alternatively, by recruiting a high fidelity DNA polymerase during the patch repair.

In order to examine if AID is able to trigger mutations in the absence of DNA replication in a non-B cell line, we developed a highly sensitive assay based on the reversion of nonsense mutations of the EGFP gene cloned in a non replicating vector. We show that the appearance of EGFP positive cells in non-B cells is dependent on the expression of AID and that, even in the absence of vector replication, mutations are found both at A∶T pairs and at G:C pairs.

## Results

**Figure 1:**
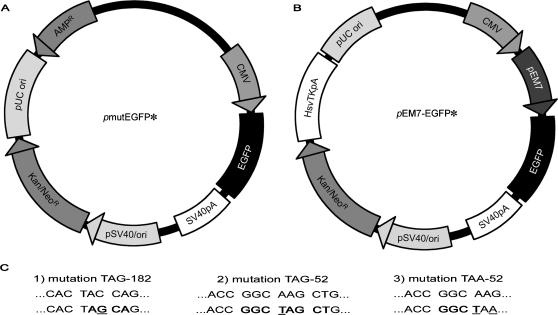
SHM vectors used to monitor SHM in AID-expressing cell lines (A) *p*mutEGFP vector: EGFP (enhanced green fluorescent protein), a variant of the GFP gene, is placed under the control of the CMV promoter (human cytomegalovirus immediate-early promoter/enhancer); pUC–prokaryotic origin of replication; AMP^R^ –ampicillin resistance gene; SV40 ori–eukaryotic origin of replication; Kan^R^/Neo^R^–kanamycin/neomycin resistance gene (B) *p*EM7-EGFP vector: EGFP is placed under the control of both an eukaryotic (CMV) and prokaryoric (EM7) promoter; pUC–prokaryotic origin of replication; SV40 ori–eukaryotic origin of replication; Kan^R^/Neo^R^–kanamycin/neomycin resistance gene (C) Sequence contexts of premature stop codons in the EGFP gene. The upper row corresponds to the wild-type sequences and the lower row to the mutated sequences. Three variants of the EGFP gene inactivated with a premature termination codon introduced by site-directed mutagenesis were used 1) The G of the TAG termination codon at the position 182 is embedded within the RGYW hotspot motif and is thus a potential target of AID 2) The TAG stop codon at position 52 lies within two RGYW motifs 3) TAA codon at position 52 is used to monitor A∶T mutations only. RGYW motifs are shown in bold letters. The mutated nucleotides are underlined.

The system we developed to study AID-dependent mutation is a simian virus 40 (SV40) -based vector containing a mutated EGFP gene to score mutations (SHM vectors: Figure 1*A and B*). We transfected the SHM vectors into cells that do not express the T antigen to prevent the plasmids from replicating, which was confirmed by a DpnI replication assay (data not shown). Because an essential component for plasmid replication is missing, only mutations associated with mismatch repair will be detected [13]. The EGFP gene was mutated by introducing a premature stop codon, TAG or TAA (Figure 1*C*). The mutated EGFP protein is truncated and non-fluorescent. If the stop codon reverts fluorescence is restored and the cells can be detected by flow cytometry in the green fluorescence channel. The number of vector molecules present in transfected cells can not be evaluated precisely thus, it is not possible to estimate a mutation rate. The mutation level is, therefore, a relative value and corresponds to the percentage of fluorescent cells. This value depends on the percentage of cells transfected with an SHM vector. Consequently, the mutation level was expressed as the percentage of fluorescent cells relative to the transfection efficiency. Transfection efficiency was estimated using a plasmid containing a wild-type EGFP gene and was typically around 30–50% in Jurkat cells, 15–35% in Jurkat-AID cells.

**Figure 2:**
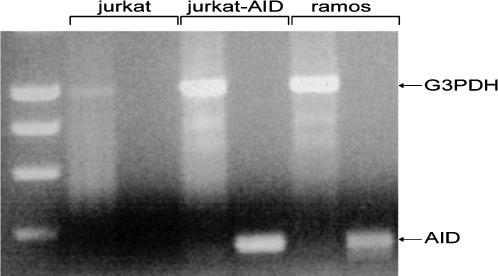
AID is transcribed in the Jurkat-AID and not in the control Jurkat cell line. AID transcription was monitored with RT-PCR in a control cell line, Jurkat, and in a Jurkat-AID cell line stably transfected with AID. A Burkitt lymphoma cell line Ramos constitutively expressing AID was used as a positive control for AID expression. G3PDH was used as an internal control. The bands corresponding to AID (380 bp) and G3PDH (1000 bp) are shown with black arrows.

SHM vectors were transfected into AID-expressing cell lines and compared to a control cell line that does not express AID. The cell lines used were a T lymphoma cell line, Jurkat, and its AID-expressing counterpart, Jurkat-AID. The expression of AID was tested by RT-PCR analyses of the Jurkat and Jurkat-AID cells (Figure 2). The Jurkat-AID clone used in this study over-expresses AID, as illustrated in Figure 2.

### AID-dependent mutations are detected in SHM vectors less than 20 hours after transfection

**Figure 3:**
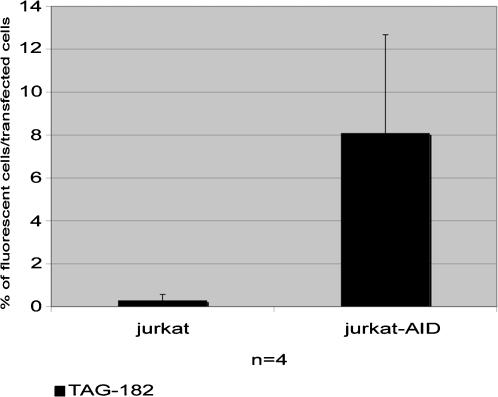
The *p*mutEGFP-TAG182 SHM vector was tested in Jurkat and Jurkat-AID cell lines. Twenty hours after transfection cells were analyzed on a FACS Scan. Cells that have reverted the stop codon of one or more copies of the vector appear fluorescent. The percentage of fluorescent cells relative to transfection efficiency was monitored using a wild-type EGFP vector. The experiment was repeated 4 times. An average of 8.1% of transfected Jurkat-AID cells reverted the stop codon, compared to 0.29% of non-transfected Jurkat cells.

To determine whether AID-induced mutations can be detected using SHM vectors, we first tested the *p*mutEGFP-TAG182 vector (depicted in Figure 1) in Jurkat and Jurkat-AID cells. As shown in Figure 3, the TAG-182 codon reverted significantly more frequently (0.3% +/− SD versus 8.1% +/−SD) in Jurkat-AID cells compared to Jurkat cells that do not express AID. To verify whether EGFP revertants are generated only by point mutations, a vector containing a 4-nucleotide deletion at position 52, which results in a stop codon, was transfected. The 4-nucleotide deletion substrate did not give rise to a functional EGFP gene in any of the cell lines transfected (data not shown). Thus, the mutations which confer the fluorescent phenotype are point mutations of the TAG stop codon. This event is AID dependent, as illustrated in Figure. 3.

**Figure 4:**
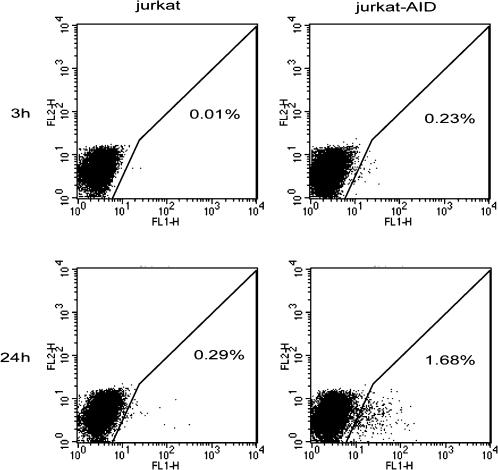
Mutations can be detected very rapidly using SHM vectors. Jurkat and Jurkat-AID cells were transfected with the *p*mutEGFP-TAG182 SHM vector. Fluorescent cells were detected 3 and 24 hours after transfection. Three hours after transfection, 0.23% of fluorescent Jurkat-AID cells are observed and 1.68% had reverted 24 hours after transfection.

In general, cells were analyzed by flow cytometry 20 hours after transfection. The maximum number of EGFP positive cells is observed between 12 and 24 hours. 24 hours after transfection 1.7% of Jurkat-AID cells reverted the TAG 182 codon. Surprisingly, we found that mutations appear very rapidly after transfection: we were able to observe EGFP positive cells within 3 hours of transfection (Figure 4). This suggests that mutations occur immediately after the DNA enters the cell.

Together these data demonstrate that AID dependent mutations can be detected with SHM vectors less than 24 hours after transfection.

### Ectopic expression of AID triggers both G:C and A∶T mutations

**Figure 5:**
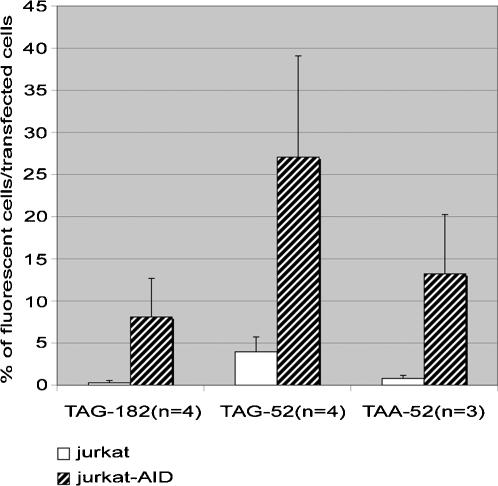
Both G∶C and A∶T mutations are detected in Jurkat-AID cells. G∶C and A∶T focused mutations were monitored using *p*mutEGFP-TAG182, *p*mutEGFP-TAG52 and *p*mutEGFP-TAA52 SHM vectors. The experiment was repeated four times for TAG-182 and TAG-52 codons and three times for the TAA-52 codon. The average value of these independent experiments is represented. The percentage of fluorescent cells was calculated relative to transfection efficiency which was monitored using a wild-type EGFP vector. Twenty hours after transfection, 8.1% of Jurkat-AID cells reverted the TAG-182 codon, 27.08% the TAG-52 codon and 13.22% the TAA codon. Less than 1% of Jurkat cells reverted TAG-182 and TAA-52 codons and TAG-52 reverted in 3.97% of the transfected cells.

In order to characterize the molecular events responsible for the introduction of point mutations in the absence of DNA replication, we constructed two new vectors in addition to pmutEGFP-TAG182 : pmutEGFP-TAG52 and pmutEGFP-TAA52 which contain different stop codons at position 52 of the EGFP gene. These SHM vectors were transfected into Jurkat and Jurkat-AID cells and analyzed by flow cytometry 20 hours after transfection, as previously described. Figure 5 shows that all the three vectors, bearing different stop codons, were mutated more efficiently in the Jurkat-AID than in the Jurkat cell line. The TAG-52 mutation reverted in both transfected cell lines, at a higher level compared to the other mutations. It reverted at a sevenfold higher level in Jurkat-AID cells compared to Jurkat cells.

In order to identify which reverse mutation confers the fluorescent phenotype, we constructed a second series of vectors, pEM7 SHM vectors, with the EGFP gene under the control of both a prokaryotic (EM7) and a eukaryotic (CMV) promoter. These vectors allowed us to extract plasmid DNA after transfection and transform bacteria to analyze individual fluorescent clones by sequencing. We transfected the pEM7 SHM vectors into Jurkat and Jurkat-AID cells.

The vectors used were *p*EM7-EGFP-TAG182, *p*EM7-EGFP-TAG52 and *p*EM7-EGFP-TAA52 (Figure 1*B and C*). The reversion efficiency of EM7 SHM vectors (data not shown) is similar to those of SHM vectors (Figure 5).

To identify the nucleotide within the stop codon that is mutated in revertants of TAG-52 and TAG-182 SHM vectors, we first sorted out fluorescent cells, then extracted plasmid DNA after secondary cloning in *E.coli.* Sequencing data demonstrate that the TAG-52 codon is mutated at the first T:A base pair (Table 1), TAG-182 is mutated at the G:C base pair in Jurkat-AID cells (Table 1). Interestingly, in the case of TAA-52, one revertant sequence bears a point mutation to TAC, thus mutated at the third A∶T base pair, instead of the expected reversion to AAA or wild-type AAG (Table 1). This encodes a tyrosine instead of a lysine and despite these aminoacid differences, this restored the fluorescent phenotype. No mutations were detected outside the stop codon in any of the 24 revertant sequences analyzed. In order to examine the overall distribution of mutations in the EGFP gene we sequenced the gene from 178 non-fluorescent colonies, 106 from Jurkat-AID cells and 72 from Jurkat cells. This analysis uncovered only two point mutations that were observed on plasmids from Jurkat-AID cells. The two mutations were the same: a G to A transition positioned near the end of the EGFP gene and seem to correspond to the same mutational event (data not shown).

**Table 1:** Reversion status of plasmids rescued from fluorescent colonies

Vector type	Cell type	Reversion sequence	G∶C or A∶T mutation
TAG-52	jurkat	…GGC AAG CTG…	A∶T
TAG-52	jurkat-AID	…GGC AAG CTG…	A∶T
TAG-182	jurkat-AID	…CAC TAC CAG…	G∶C
TAA-52	jurkat-AID	…GGC TAC CTG…	A∶T

## Discussion

In the present study we made use of non-replicating episomal vectors to study AID induced mutagenesis in a non-B cell context. Our system is based on the reversion of a nonsense mutation in an EGFP gene, cloned downstream of the CMV promoter/enhancer. The data demonstrate that transiently transfected DNA can be mutated in a AID dependent manner in non-B cells. Both G:C and A∶T mutations were detected, suggesting that the lesion introduced by AID is sufficient for triggering both types of mutations. The high sensitivity of our transient assay is probably due to the high plasmid copy number introduced in each cell (10^5^-10^6^ per cell) and to the fact that the reversion of only one stop codon per cell is sufficient to be detected on a FACS analyzer.

In general, phase 1 mutations, located at the level of the U∶G mispairs, are only found after the replication fork has passed the abasic site produced by uracil-DNA glycosylase and a dNTP has been inserted opposite the abasic site. The fact that the reporter gene can not replicate demonstrates that, not only G:C, but also A∶T mutations are not typical phase 1 mutations.

How can we explain the mutability of the reporter gene in the absence of DNA replication in Jurkat-AID cells? Numerous studies have shown that the rate of mutation of V regions is proportional to the rate of transcription. The biochemical demonstration that AID deaminates C in single stranded (ss) DNA led to the proposal that transcription triggers separation of the DNA strands, each ss DNA is then exposed to the action of AID [6], [14]–[17]. The EGFP reporter gene is under the control of a strong promoter and this could explain its high mutability, caused by the formation of ss DNA created by the supercoiling of the DNA. It is interesting to observe that all three types of codons were reverted in the Jurkat-AID cell line and that the TAG-52 codon also reverted at a significant level in Jurkat cells without AID. The high mutability of the TAG-52 codon can be explained by the influence of secondary structures. Wright and coworkers showed that the position of the nucleotide within the stem loop structure determines the mutability of the nucleotide in prokaryotes [18] and in eukaryotes [19], [20]. The most hypermutable bases are located immediately next to stems in stable DNA stem-loop structures (SLS). In light of this, we can assume that the background mutation for the TAG-52 codon, which lies within two adjacent hotspot motifs, is considerably higher compared to the other codons, due to a secondary structure effect (SLS effect). This phenomenon is amplified in AID expressing cells.

How can the formation of a U∶G mismatch by AID trigger the reversion of the stop codons? Theoretically the elimination of a mismatch in non-dividing cells by MMR can operate without distinguishing between the two DNA strands [21]. In the case of a U∶G mismatch this results, with equal probability, either in a G∶C → A∶T transition or in the maintenance of the sequence. Clearly none of the reversions of the three stop codons in the EGFP gene correspond to a G∶C → A∶T transition. We therefore postulate that the reversion of the stop codon is introduced during MMR. MMR involves the recognition of the U∶G mismatch by the Msh2/Msh6 heterodimer, endonucleotide cleavage of one of the DNA strands and the creation of a gap by exonuclease I. While gap repair is usually error-free due to the activity of high fidelity DNA polymerase δ & ε [12], [22], during phase 2 of SHM, the gap could be repaired by error-prone DNA polymerases that insert mispaired nucleotides at A∶T pairs [10], [23]. A key question is why the mechanism that normally insures the fidelity of DNA repair in non-B cells seems ineffective in Jurkat-AID cells? DNA mismatch repair is normally used to correct mispairing occurring during DNA replication. Its efficiency is based on its ability to distinguish between parental and neosynthetized DNA strands. In the absence of DNA replication a mismatch will be repaired with equal probability to fix the mutation or to restore the wild type sequence. If theorically mutagenic mismatch repair in the absence of DNA replication does not require a specialized DNA polymerase, the high rate of mutation observed during SHM is achieved by the recruitment of specialized error-prone DNA polymerase [23], [24].

These results strongly support the view that, even in non dividing cells, mismatch repair can trigger mutations at distance from the initial mismatch. In addition, phase 2 mutations can be expressed independently of phase 1 mutations, before the passage of the replication fork.

## Materials and Methods

### Plasmid constructions

*p*mutEGFP-TAG182, *p*mutEGFP-TAG52, *p*mutEGFP-TAA52 were obtained as follows. First, an AseI MluI fragment from the pEGFP-C1 plasmid (Clontech) was inserted into an XhoI site (after Klenow fragment (KF) treatment) of the pBluescript sk+ plasmid (Stratagene) to create psK+EGFP. A Bsu36I fragment from the pEGFPC1 plasmid was cloned into an EcoRV site of the pSK+EGFP vector to obtain the pEGFPcontr vector. This vector was mutated by the site-directed mutagenesis kit (Stratagene) using the following primers: TAG-182 mutation, pTAG182a (gctcgccgaccactAGCAgcagaacaccccc) and pTAG182b (gggggtgttctgcTGCTagtggtcggcgagc); TAG-52 mutation, pTAG52a (catctgcaccaccGGCTagctgcccgtgccctg) and pTAG52b (cagggcacgggcagctAGCCggtggtgcagatg); TAA-52 mutation, pTAA52a (catctgcaccaccGGCTaActgcccgtgccctg) and pTAA52b (cagggcacgggcagTtAGCCggtggtgcagatg).

The second series of vectors enables the transcription of EGFP in both eukaryotic and prokaryotic cells. pEM7-EGFP is based on the pEGFP-C1 vector (Clontech). pEGFP-C1 was digested with NheI and XhoI and recirculized to obtain the P1 vector. The EM7 promoter was obtained by annealing 2 oligos EM7EcoRI/AfeI 1 (aattcTGTTGACAATTAATCATCGGCATAGTATATCGGCATAGTATAATACGAAGGTGAGGAACTAAAccatgagcgct) and 2 (agcgctcatggTTTAGTTCCTCACCTTGTCGTATTATACTATGCCGATATACTATGCCGATGATTAATTGTCAACAg) (phosphorylated at the 5′ end). The pEM7 plasmid was obtained after introduction of the EM7 promoter into EcoRI and SmaI digested P1. pEM7 was digested with AfeI and BamHI. The EGFP insert with a 3′ BamHI site was prepared by PCR of pEGFPcontr, pmutEGFP-TAG182, pmutEGFP-TAG52 and pmutEGFP-TAA52 using the following primers: 5′GFP (aagggcgaggagctgttcaccG) and 3′GFP (AGGGTAGGATCCcttgtac agctcgtccat). A BamHI site was inserted into the 3′ primer. The PCR product was digested with BamHI and cloned into the pEM7 vector in order to obtain pEM7-EGFPcontr, pEM7-EGFPTAG182, pEM7-EGFPTAG52 and pEM7-EGFPTAA52 vectors.

pCDNA3.1AID was constructed as follows. AID cDNA was obtained from a cDNA library of Ramos cells (produced using the Creator SMART cDNA Library Construction Kit from Clontech) and initially cloned into the pCDNA-LIB plasmid (Clontech). AID cDNA was amplified by PCR and then transferred into the pET28 plasmid (Novagen) using NheI and XhoI sites, pCDNA3.1AID was obtained by cloning AID in the NheI and XhoI sites of pCDNA3.1HisA (Invitrogen).

### Cell lines

The Jurkat cell line is a T lymphoma cell line that does not express AID. Jurkat-AID cell lines were obtained by transfection of the Jurkat cell line with pCDNA3.1AID. 10 µg of plasmid was used for transfection of 1×10^7^ cells by electroporation. 48 hours after transfection G418 (neomycin) was added for selection at a final concentration of 2 mg/ml and cells were distributed in three 96 well plates at a concentration of 3 cells/well. After approximately three weeks of selection, clones were obtained, amplified and tested for AID expression by RT-PCR (see below).

### Cell culture and transfection

Jurkat and Jurkat-AID cell lines were cultured in RPMI glutamax, with 10% FCS, 100 units/ml penicillin and 100 µg/ml streptomycin, and 2 mg/ml G418 for the Jurkat-AID cell line at 37°C, 5% CO_2_. In transfection experiments 25 µg of SHM vector DNA were introduced by electroporation into 1×10^7^ Jurkat and Jurkat-AID cells in 0.4 cm cuvettes using a Biorad Gene pulse electroporator. The conditions used were: 260 V, 975 µF, R = ∞. After transfection, cells were resuspended in 10 ml of fresh medium and cultured at 37°C, 5% CO_2_ for 20 h (except when otherwise indicated). As a transfection control, 25 µg of plasmid expressing wild-type, fluorescent EGFP were transfected.

### Flow cytometry

Twenty hours (except when otherwise indicated) after transfection, 5 ml of transfected cells were centrifuged and resuspended with PBS, 0.5% FCS, 2 mM EDTA, 0.5 µg/ml propidium iodide (dead cell marker) and analyzed by flow cytometry on a FACS Scan (BD Biosciences). The acquisition was carried out on 500 000 cells. Analysis of the acquired data was performed with the «Cell Quest» software (BD Sciences, Mountain View, CA). For plasmid sequencing, fluorescent cells were sorted on a Moflo cell sorter (DakoCytomation) before extraction in order to concentrate fluorescent colonies (see below).

### Extraction of plasmid DNA from mammalian cells

The NucleoSpin Plasmid (Macherey Nagel) kit for extraction of plasmid DNA from bacteria was adapted to extract plasmid from mammalian cells. Twenty hours after transfection 5 ml of transfected cells were washed with PBS, centrifuged and subjected to extraction. After resuspension and lysis (according to manufacturer's instructions), the material was treated with 800 µg/ml proteinase K for 1 h to 2 h at 55°C. Proteinase K digestion was followed by neutralization, column fixation, washing and elution (according to manufacturer's instructions). In the DNA replication assay plasmid DNA was digested with DpnI 2 h at 37°C.

### Transformation in *E.coli* and sequencing

2 µl of extracted DNA was transformed in TOP10 bacteria (Invitrogen) and resuspended in 900 µl of SOC medium. The total suspension was plated on LB kanamycin (50 µg/ml) plates, 100–200 µl of bacteria suspension per plate. Plates were analyzed on a Lighttools Illuminatool Tunable Lighting System. Fluorescent colonies were grown in 4 ml LB kanamycin medium overnight. Plasmid DNA was extracted using NucleoSpin Plasmid (Macherey Nagel) kit according to manufacturer's instructions and sent for sequencing using a CMV primer (gtacggtgggaggtctatataagcag).

### AID RT-PCR

Total RNA was extracted from Jurkat, Jurkat-AID and Ramos cells using the Trizol reagent (Invitrogen) according to manufacturer's instructions. Two µg of RNA was denatured 5 minutes at 65°C with 0.5 µg of oligo dT and chilled on ice. Reverse transcription was performed in a 50 µl reaction for 90 minutes at 37°C with 1xMMLV buffer, 0.5 mM dNTPs, 10 mM dithiotreitol, 200 U of MMLV (all from Invitrogen) and 400 U/ml RNAsin (Promega). The MMLV was inactivated at 70°C for 15 min. 2 µl of the cDNA was used for each PCR reaction: AID and G3PDH. PCR was conducted using Taq polymerase (Qiagen), according to manufacturer's instructions, in a 50 µl reaction using 0.4 µM of the following primers: AID1 (TAGACCCTGGCCGCTGCTACC) and AID2 (CAAAAGGATGCGCCGAAGCTGTCTGGAG) for AID amplification, G3PDH1 (TGAAGGTCGGAGTCAACGGATTTGGT) and G3PDH2 (CATGTGGCCATGAGGTCCACCAC) for G3PDH amplification. The cycling used for both PCRs is 94°C/2 min, 15 cycles of 94°C, 15s; 65°C, 30s; 72°C, 45s, 30 cycles of 94°C, 15s; 65°C, 30s; 72°C, 45s+5s every cycle and 7 minutes at 72°C.
